# Emergency medicine in Japan: past, present, and future

**DOI:** 10.1186/s12245-020-00316-7

**Published:** 2021-01-07

**Authors:** Kentaro Shimizu, Seikei Hibino, Michelle H. Biros, Taro Irisawa, Takeshi Shimazu

**Affiliations:** 1 Department of Traumatology and Acute Critical Medicine, Osaka University Graduate School of Medicine, 2-15 Yamadaoka, Suita-shi, Osaka, 565-0871 Japan; 2 Department of Emergency Medicine, University of Minnesota, 717 Delaware Street SE, Minneapolis, MN 55455 USA

**Keywords:** Acute medicine, Emergency medicine, Japan, Ambulance diversions, Elderly, Education

## Abstract

Quite a few changes and challenges have arisen in society in general as technology has advanced and the aging population has increased. These can lead to the recognition of the shortcomings of a society’s traditional systems and the various changes that are needed, especially in providing emergency medical care. A super-aged society has been developing in Japan, and the emergency care system needs to change according to these new demographics and society’s needs. The focus has been shifting from critical care and trauma to medical and surgical conditions involving the elderly. Challenges in triage, ambulance diversion, and staffing are discussed in this review. Possible solutions currently underway, such as a public helpline, smartphone app system, coordination by designated hospitals, and universal coverage/government support, are discussed as future directions. Emergency medicine in Japan needs to develop in a more flexible way to meet the upcoming robust challenges of the changing demographics.

## Background

There have been no recent reviews on the status of the development of emergency medicine in Japan [1, 2]. Over time, the demographics of Japan have changed drastically as the population has grown older and the birth rate has steadily declined. In fact, Japan has the largest population of people older than 65 years (26.3%) according to the 2018 worldatlas.com [3]. The kinds of emergencies seen in Japanese hospitals have also changed as the safety features of automobiles have improved.

The traditional method of providing emergency care in Japan’s large metropolitan areas is organized according to three levels of emergency depending on the perceived acuity of the patient as evaluated by paramedics. The idea behind this structure was to centralize the transfer of the sickest patients to tertiary emergency centers to optimize their care. However, recent changes in the demographics of the population and the types of injuries sustained have considerably challenged this traditional system. In this review, emergency medicine in Japan is described by reviewing its history, recent developments and current structure, present challenges, and future directions in a country facing an increasingly aging demographic.

### History and the changing demographic of emergency patients

**Fig. 1:**
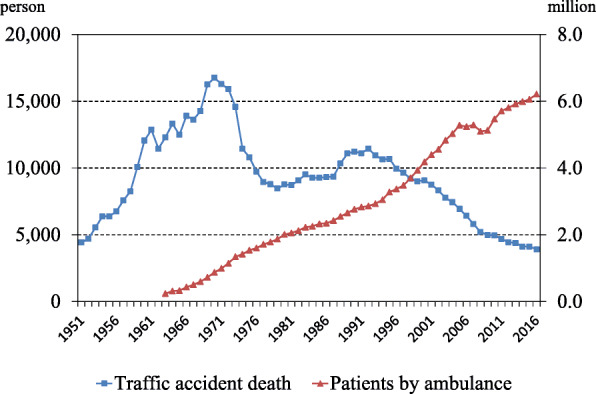
Annual numbers of deaths by traffic accident and patients transported by ambulance. As the population in Japan grows older and more effective medical treatment is developed, ambulance use is expected to increase

From the 1930s, local governments hired paramedics and provided ambulance service to hospital emergency departments (EDs) at no cost to the general public [2]. In the 1960s, the number of trauma patients in Japan rapidly increased as the economy developed. By 1970, the number of deaths by traffic accidents was reported to be 16,765 [4]. At that time, emergency patients were seen by physicians of different specialties according to the perceived medical need. Difficulties in management arose when a trauma patient sustained multiple injuries and required simultaneous care from more than one specialty. Instead of providing simultaneous trauma care, care was delivered sequentially by a series of specialists sometimes at different facilities. As a result, the injured patients were diverted multiple times to provide specialty care for all of their needs. In 1967, the Osaka University Critical Care Center was founded to provide all necessary care for patients with multiple trauma at one location for the first time in Japan. After 1971, the number of trauma patients decreased after the law required the use of seatbelts for cars and helmets for motorcycles and decreased even more after the institution of a “no drink and drive” rule (3904 fatalities/year in 2016). At the same time, the number of designated tertiary emergency/critical care centers increased throughout Japan to 289 centers in 2018 [5]. In contrast to the decreased numbers of trauma patients, the number of ambulance activations increased and was up to 6.2 million in 2018 [6] (Fig. 1). Disease categories of the patients in emergency centers in 2011 consisted of trauma (18.3%), stroke (17.3%), cardiovascular disease (15.5%), digestive disease (11.0%), cardiac arrest (9.6%) and others [7]. These results suggest that causes of ambulance activation have changed from young trauma patients to older medical patients. As the population in Japan grows older and medical interventions in the emergency field develop, ambulance use is expected to increase. Additionally, the overuse and abuse of the free ambulance service is also being recognized as a problem.

### Recent developments and current structure

#### Emergency medical system

Emergency medical services (EMS) are provided through a 1-1-9 telephone number designated as the universal emergency access number that directly connects to the dispatch center located in the regional fire defense headquarters. The nearest available ambulance is sent to the incident. All expenses are covered by local governments, and there is no charge to the patient for care and/or transportation. EMS training is stratified into three levels of pre-hospital emergency care personnel: basic-level ambulance crew, personnel with an intermediate level of expertise (SFAC [Standard First Aid Class]), and those with an advanced level (ELST [Emergency Life-Saving Technician]). Ambulance personnel eligible for ELST must have 5 years or 2000 h of experience as SFACs [8].

**Fig. 2:**
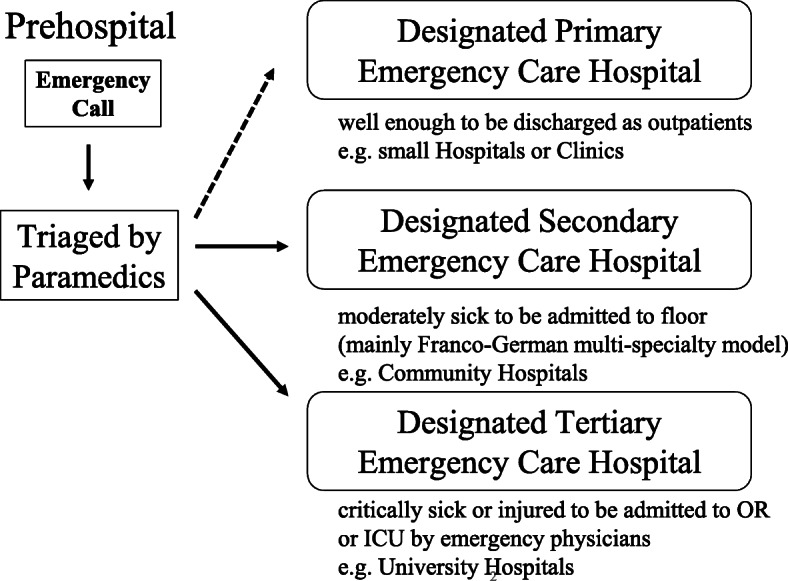
Prehospital Triage System depending on acuity in Japan. Emergency medical service personnel respond to emergency calls and triage patients according to their perceived acuity per the protocol. A Designated Primary Emergency Care Hospital is chosen if the patient is well enough to be discharged. A Designated Secondary Emergency Care Hospital is chosen if the patient is sick enough to be admitted to a Medical/Surgical Floor. A Designated Tertiary Emergency Care Hospital is chosen if the patients is sick enough to be admitted to the Intensive Care Unit (ICU) or to require emergent interventions in the operating room (OR)

There are three designated levels of emergency hospitals in Japan, which are categorized according to the perceived acuity of the patient (Fig. 2). A designated “primary” emergency center deals with patients who can be managed as outpatients, a designated “secondary” emergency center deals with patients who can be managed as inpatients on a general medical floor, and a designated “tertiary” medical center deals with patients who need to be managed in the operating room or the ICU [9]. This system has provided the Japanese public with efficient, high-quality medical care as reported in 1985 [10]. The paramedics triage patients at the scene and transport them to the most appropriate level of the hospital. Their triage decisions are reviewed periodically at medical control conferences conducted in each city.

Patients with immediately lethal conditions such as cardiac arrest, multiple trauma, and stroke are transferred directly to designated tertiary emergency hospitals. “Walk-in” patients are only seen in the designated primary and secondary emergency hospitals. The designated tertiary emergency hospitals accept patients delivered by ambulance but do not accept walk-ins. The idea here is to centralize care for the most critical patients and optimize resource utilization. For example, several tertiary hospitals implemented a new workflow concept termed the Hybrid ER, which comprises a computed tomography (CT) scanning system with interventional radiology features that allow CT examination and emergency therapeutic intervention in the same room without relocating the patient. This means that the first bed for triaged critically ill patients is the CT table. The Hybrid ER system has decreased mortality in the ER [11, 12].

Pre-hospital triage by emergency medical personnel (all public employees) maintains the quality of emergency care stratified to the best of their knowledge. The number of hospital beds in Japan is the highest in the world (12.98 [Japan] vs. 2.87 [USA] per 1000 inhabitants) [13]. Yet at the same time, diversions to other hospitals often occur. As elderly patients and those with loss of consciousness involve broad differential diagnoses, it would be quite challenging and difficult for medics to triage patients to an ED with “appropriate staff.” This is a common scenario causing “out of their specialty” diversions and has resulted in long, unfortunate ambulance diversions. The primary reasons for the diversions include elderly patients, foreigners, patients with loss of consciousness, nighttime, and weekend/holidays [14].

In the era of coronavirus infection (COVID-19) in 2020, there has been an increase in the degree of difficulty in gaining hospital acceptance due to acute disease in 2020 compared to that in the same week in 2019 despite the decrease in the number of transported patients [15]. However, because triage is performed by EMS personnel prior to patient arrival at the emergency room (ER) and does not increase waiting time in the ER, the potential for overcrowdedness in the ER is reduced.

### Certification/staffing

#### Multi-specialty staffing and insufficient numbers of emergency physicians

The Japanese Association of Acute Medicine is the Acute Medicine/Emergency Medicine specialty board certifying body and at the same time the largest emergency medical professional organization in Japan. As of January 2018, its members numbered 10,581, and the number of board-certified members was 4790 [16]. In contrast, over 25,000 emergency physicians are board-certified by the American Board of Emergency Medicine, and there are roughly 35,000-40,000 emergency physicians practicing in the USA [17]. The focus of board certification in Japan is somewhat different from the emergency medicine certification in the USA.

Emergency physicians working in the designated tertiary emergency hospitals usually have training in one additional specialty, such as Trauma Surgery, Interventional Radiology, or Critical Care in Japan. This may be because for the most part, Japan operates a multi-specialty staffing model in metropolitan areas, unlike the single-specialty staffing in the US EDs. In the designated secondary emergency hospitals, internists and surgeons frequently work as moonlighters for night ED coverage. Most patients in designated primary and secondary emergency hospitals are treated by specialists other than Acute Medicine/Emergency Medicine physicians. This has been necessary for some hospitals to help alleviate staffing issues. As one can imagine, lifelong education for these doctors such as internists and surgeons is necessary to maintain high-quality emergency care.

### Changes in the postgraduate training system and influence on the emergency system

In 2004, the postgraduate training system in Japan changed, and a 2-year Preliminary Clinical Training course (with 3 months set aside for an elective class in Emergency Medicine) became mandatory [18]. The main purpose of this system was to provide primary care training before specialty training and to provide an adequate salary to the trainees. In essence, the Ministry of Health, Labor and Welfare hoped to better prepare for an aging population’s medical needs [19]. Postgraduate trainees frequently used to moonlight in EDs in smaller community hospitals or clinics to increase their income as they were not paid well enough. This new system meant that there was no further need for postgraduate trainees to staff smaller community hospitals’ EDs through moonlighting as was done commonly in the past. As a result, a number of hospitals relinquished their emergency hospital designation because of staffing issues at night. The number of designated emergency hospitals thus decreased from 4965 in 2004 to 4370 in 2008 [20, 21].

In 2008, it was reported that designated tertiary emergency centers refused patients and diverted ambulances about 200 times a year on average. Then, the tragic case of a pregnant woman who died of intracranial hemorrhage after multiple ambulance diversions was widely reported in 2006. Nineteen EDs refused to accept this patient for various reasons, and it took about 3 h for her initial evaluation [22]. These unfortunate ambulance diversions were due not only to short staffing but also to the system of multi-specialty model staffing, which requires more staffing and provides more reasons for patients to be diverted because their condition is “outside of their specialty,” and no specialist was available in the hospital. Adoption of the single-specialty staffing model of emergency physicians in the Anglo-American system may solve this issue to some extent, and this has begun to occur in some parts of Japan [23].

#### Elective postgraduate training after preliminary training years

The 2-year Preliminary Clinical Training program has helped to standardized the education system to assist trainees in learning primary care medicine. Trainees rotate through several specialties for 1 to 3 months. After 2 years, they can specialize in their specialty of choice. In the third year, they join their specialty residency training for 3–4 years. What commonly occurs after that in the Osaka University Program is for the physicians to study for a PhD degree. Usually, they do basic research and part-time clinical work during this 4-year period.

In our program, preliminary-year trainees (PGY 1 and 2) work in several departments but have no opportunities to see all types of acuity and varieties of disease. First of all, although emergency medicine is a required field, the residents’ required rotation can be done partially with anesthesiology or critical care medicine. Second, within the current emergency medical system, patients are triaged depending on their perceived acuity and transferred to designated emergency centers of different levels. Preliminary-year trainees may not see enough patients of different acuity levels to learn emergency medicine comprehensively at one designated tertiary emergency hospital where only most critically ill or injured patients are seen. It is highly desirable for them to gain skills in recognizing high-acuity conditions that can develop from seemingly low-acuity conditions.

A more standardized program is needed so that trainees can rotate through not only designated tertiary emergency care centers but also designated primary and secondary emergency hospitals to gain comprehensive experience.

### Aging population and emergencies

#### World’s highest aging population

One of the biggest reasons for the increase in ambulance activations in Japan is due to its aging population. Japan has the highest proportion of elderly people in the world with 26% of the population (2018) over the age of 65 years (14% in the USA). The average health life expectancy was 70.4 years in males and 73.6 years in females in 2010 [24], and in 2016, the life expectancy in Japan was one of the highest in the world (males: 81.0 years; females: 87.1 years). Among people aged more than 65 years in 2016, 27.1% lived in single-person households [25]. This suggests that most people will require support from others for about the last 10 years of their life. In contrast, the population of the younger generation has been decreasing in Japan. The total fertility rate in Japan was only 1.44 in 2016.

#### Community comprehensive care system

**Fig. 3:**
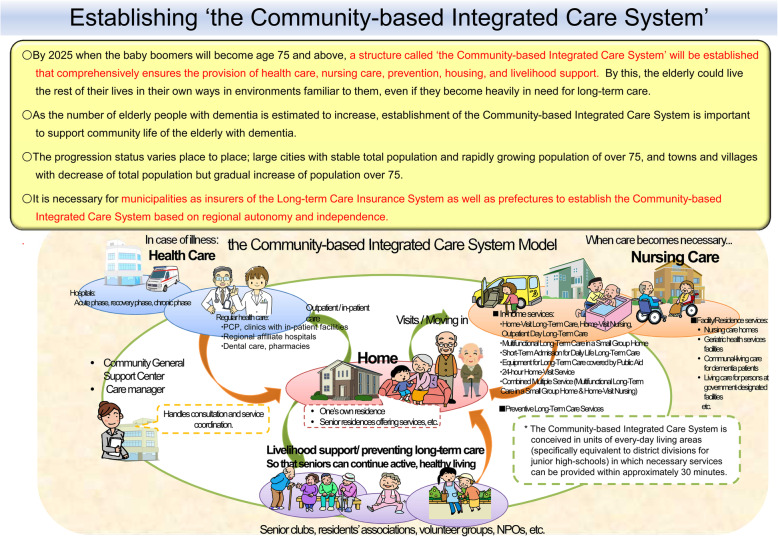
The community-based Integrated Care System Model. By 2025, when the baby boomers will reach the age of 75 years old and older, a structure called “the Community-based Integrated Care System” will be established that comprehensively ensures the provision of health care including emergency care, nursing care, prevention, housing, and livelihood support. The elderly will be able to live the rest of their lives in familiar environments and be accommodated for their long-term care needs [27, 28]

The government established a policy for an integrated community system that includes residential arrangements, living arrangements, medical prevention, medical care, and nursing care for elderly patients so that they are able to stay in the community they are familiar with [26] (Fig. 3). Many patients may have to change their place of residence depending on their medical needs, moving from home to assisted-living center to nursing home, but the integrated community system provides them with a way to remain in their community through a smooth transition to the required level of care. What has not been discussed in this integrated care system is when to activate emergency care for patients at the end of life. As illustrated in Fig. 3, there is no budgetary support available for emergency or end-of-life care. The quality of out-of-hospital medical care can be more standardized and can be audited. Although ambulance activations are expected to increase with the increasingly aged population, the establishment of this community comprehensive care system and further discussion of end-of-life care may alleviate futile ambulance activations and unburden the Emergency Medical System.

#### Transfer of elderly people in nursing homes in emergencies

Japanese people die at hospitals (80.3%), at home (12.6%), and in nursing homes (4.8%) [29]. However, only 17.9% of people would like to die in the hospital. About 60% take the family’s burden into account when they think about a desirable place to die. There are discrepancies between people’s wishes and reality; however, elderly people in nursing homes usually do not want to be transferred to intensive care at the end of life. Kitamura et al. examined elderly patients with bystander-witnessed out-of-hospital cardiac arrest in Osaka [30]. Among these patients, most came from their home (70.4%, 7656/10,876) or from a nursing home (12.9%, 1358/10,876). One of the reasons for the number of patients from nursing homes is that they frequently do not require and therefore sometimes do not have advanced directives on handling patient emergencies. The employees and caregivers have to decide whether to call for emergency paramedics, which may well be futile. There are no mandatory rules to write medical advance directives such as POLST (Physicians Orders for Life-Sustaining Treatment) or MOLST (Medical Orders for Life-Sustaining Treatment) when elderly people are admitted to nursing homes [31]. This is an area requiring cooperation with legislative side of government, although such rules can be developed voluntarily at each institution.

#### Suicide

In Japan, suicide is the leading cause of death among people aged between 15 and 39 years. The main methods are by hanging (> 50%), jumping from a height, and overdosing on drugs. The number of suicides increased after the recession in 1998 from 24,391 to 32,863 and has remained at around 30,000/year despite passage of a suicide prevention act [32]. After the Ministry of Health, Labor, and Welfare Special Committee on Prevention of Suicide released its final report on national suicide prevention strategies, the number of suicides decreased to 21,897. About 40% of those committing suicide were older than 60 years. The suicide rate in Japan is among the top 6 in the world [33]. Emergency physicians have an important role in connecting attempted suicide patients with mental health professionals.

### Future directions and current solutions: a prehospital system to avoid ambulance diversion (Fig. 4)

#### Twenty-four-hour helpline

Out-of-hospital healthcare is essential in providing effective healthcare to patients. The National Health Service Direct was first established in 1998 to provide 24-h/7-day-a-week nurse-led telephone-based healthcare advice and information to the public in England and Wales [34]. In some cities such as Osaka and Tokyo, the 24-h helpline center “*Anshin*” (peace of mind) is available [35, 36]. Telephone triage and 24-h helplines like those in the UK could possibly decrease the number of ambulance activations and ambulance diversions, although a similar service might have increased the number of ambulance activations in the USA, possibly due to its litigious environment.

**Fig. 4:**
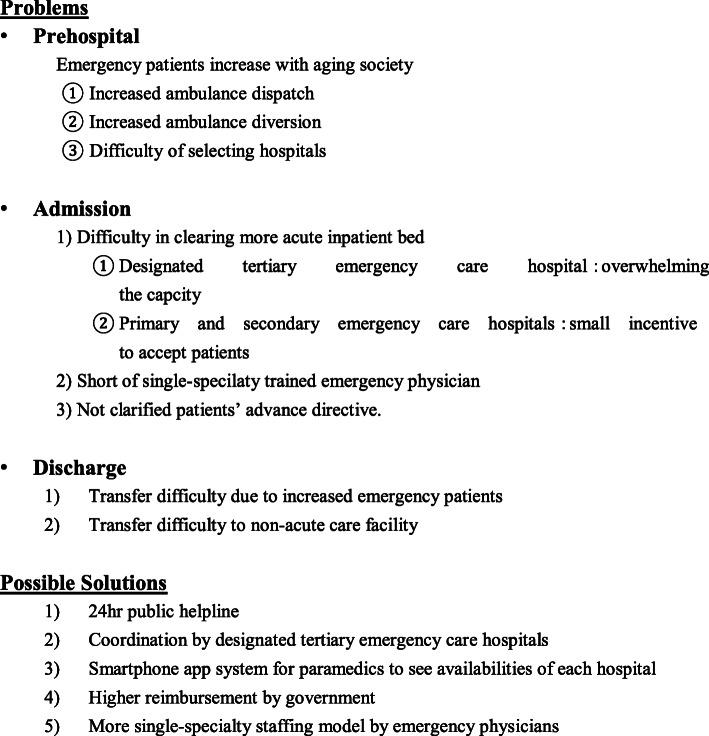
Problems and possible solutions of emergency medical care in a super-aged society

#### Protect Net by designated tertiary emergency hospitals

Protect Net (“*Mamotte Net*”) is a coordination program by designated tertiary emergency hospitals. When paramedics determine that a patient needs to be triaged as high acuity but the transport time is expected to be more than 30 min or that 5 different EDs will not accept the patient, the fire department can request transfer to a different designated emergency hospital through an online network designed to coordinate an appropriate hospital transfer. The designated tertiary emergency hospitals are required to accept the patients for stabilization and then decide further disposition. This program acts as a safety net in the Osaka metropolitan area.

#### Smartphone EMS app for paramedics and the public

The medical information system in Osaka called ORION (Osaka emergency information Research Intelligent Operation Network system) was developed to share information between an ambulance and a hospital by using a smartphone app at the scene. This smartphone app system was introduced into the EMS system in 2013 to facilitate hospital selection and the transport of emergency patients. EMS personnel input the time and place of the incident, patients’ vital signs, symptoms, and other information. They can share information across appropriate hospitals depending on acuity and presumed disease and treatment among themselves and determine the most appropriate hospital to accept the ambulance using a GPS system and ORION [37, 38]. The rate of difficulty in obtaining hospital acceptance, which was defined as EMS personnel at the scene making ≥ 5 phone calls to hospitals, significantly decreased after the introduction of this smartphone app. For the public, there is also a smartphone app to instruct them on whether to call ambulance, which was produced by the Fire and Disaster Management Agency.

#### Universal coverage support for emergency medical care

Medical expenditures account for 10.3% of the GDP in Japan compared with 16.9% in USA [39]. The number of practicing physicians is 17.1 per 100 beds compared with 85.2 per 100 beds in the USA [40]. The average hospital in-patient stay is 16.5 days in Japan but is only 6.1 days in the USA [41]. Medical expenditures and the number of doctors have been strictly regulated by the government to ensure universal coverage [42]. Medical costs in Japan are regulated by universal coverage. For example, 70% of the cost is paid by the government for adult less than 70 years old [43]. The maximum burden of expense is set in accordance with income [40].

The Ministry of Health, Labour and Welfare has revised the payment system for emergency medical care under universal coverage. To improve the poor acceptance of pediatric, obstetric, and psychiatric emergency patients, support from the government has been provided, and the hospitals handling these kinds of emergencies are receiving government subsidies. Also, to ensure the smooth transfer of patients from acute-care hospitals to rehabilitation facilities or Transitional Care Units, the government will subsidize the process to improve output (availability of inpatient beds) [44].

#### Developing a single-specialty staffing model

An aging population and technological advances have prompted change in providing emergency care in Japan. The current Emergency Medical System, which is focused on critically ill or injured patients, needs an additional system to complement the current system to better prepare for the increasing elderly population. Although the idea that critically ill and injured patients should go to a designated tertiary emergency care center is very similar to that of trauma centers in the USA to centralize and optimize care, it has been recognized that Japan’s multi-specialty staffing model has some limitations. It is also well documented that elderly patients tend to have subtle symptoms and signs including changes in vital signs even though they are suffering from life-threatening illness or injury [45]. In relation to geriatric medicine, other countries with aging populations have successfully implemented various changes to their emergency healthcare systems. A geriatric emergency education program has already been set up in the USA [46]. The International Federation for Emergency Medicine listed eight minimum standards involving the right approach, personnel, environment, decision making, processes, support, results, and system to guide the care of the elderly in EDs and national health systems across the globe [47]. The geriatric ED intervention in Australia has been reported to reduce time to discharge and length of stay in the ED [48].

Despite the differences in cultures and healthcare systems, the development of a single-specialty staffing model in Japan has been ongoing to narrow this gap. As Okinawa prefecture was under the US occupation until 1968, they have a single-specialty staffing model equivalent to that in the US Fukui prefecture has leaders that started this model roughly 30 years ago, and it is now well recognized and respected. Tokushukai, a large healthcare community group, operates large community hospitals that have been quite active and successful. Their most recent success was at the Tokyo Bay Medical Center in the eastern part of the Tokyo metropolitan area, which was led by the US-trained emergency physicians, hospitalists, and intensivists. Tokyo now has three “Tokyo ER” facilities in their large community hospitals, although they are staffed by surgeons, internists, and pediatricians, and thus, this is not a single-specialty but an oligo-specialty staffing model [49]. Larger community hospitals are leading the way, but academic and university hospitals are also catching up. Osaka University began a program in 2013 to invite the US-trained emergency physicians to teach at their affiliated hospitals. The Japanese Association for Acute Medicine also recognized such needs and started “the ER committee” in 2004 and also “the Resident and Medical Students committee.” Their last general assembly in Osaka in 2017, with the main theme of “Love EM,” included a video to attract younger physician to emergency medicine. The requirement of 2 years of preliminary clinical training was at least partially aimed at improving primary care needs in Japan. Anecdotally, the quality of primary care has improved considerably ever since, but it is not clear what kind of impact this has had on the staffing for emergency care overall.

## Conclusions

As the country with the largest ratio of its population over 65 years of age and with highly advanced technology, the development of emergency medicine in Japan may be of great interest to other countries. To improve pre-hospital care, 24-h telephone lines, an Internet site, and fire department/EMS-operated policies are in operation as described. The government is leading the policy for end-of-life care for patients, but advance directives need to be clarified and put in place as a guideline. Japan may learn from various aspects of these changes as well. The acknowledgment of the need for increased cross-talk between different systems is also required.

The lack of available inpatient beds can be a problem, and this is sometimes a reason for hospitals not accepting an ambulance. Clearly, the coordination of acute-care hospitals with chronic rehabilitation care facilities is crucial in this regard to improve output.

To address systemic limitations, a system that establishes a multi-specialty staffing model in designated tertiary emergency care centers and the development of a single-specialty staffing model in the designated primary and secondary emergency care centers, or possibly even in the tertiary emergency care centers, will probably be required. This systemic change will then reflect the needs of Japan’s super-aged society.

## Data Availability

Not applicable

## Abbreviations

CT: Computed tomography
ED: Emergency department
EMS: Emergency medical services
ER: Emergency room
MOLST: Medical Orders for Life-Sustaining Treatment
ORION: Osaka Emergency Information Research Intelligent Operation Network system
POLST: Physicians Orders for Life-Sustaining Treatment
